# Supplementary material for: MetaRibo-Seq measures translation in microbiomes
Source: Nat Commun. 2020 Jun 29;11:3268. doi: 10.1038/s41467-020-17081-z (PMC7324362; doi:10.1038/s41467-020-17081-z)
Supplement: Supplementary file 10 — Supplementary Data 7 [file 41467_2020_17081_MOESM10_ESM.zip › File2/Confidence_High_Taxonomy/31640_out.krona.html]

Javascript must be enabled to view this page.

members
magnitude
magnitudeUnassigned
count
unassigned
taxon
rank

31640\_out

47

47
2
superkingdom

47
phylum
1239


SRS015890\_contig\_number\_11109SRS023526\_contig\_number\_26286SRS047014\_contig\_number\_28331SRS146812\_contig\_number\_51558SRS893270\_contig\_number\_11543
2292896
species
5

186801
class
39

order
186802
39

family
31979
39

1485
genus
39


SRS013215\_contig\_number\_contig-100\_278.278SRS014235\_contig\_number\_contig-100\_173.275751SRS014683\_contig\_number\_18263SRS014736\_contig\_number\_contig-100\_98.99SRS014923\_contig\_number\_24336SRS014979\_contig\_number\_contig-100\_9545.54253SRS015578\_contig\_number\_23018SRS017191\_contig\_number\_contig-100\_9311.9311SRS017521\_contig\_number\_8561SRS018427\_contig\_number\_contig-100\_1888.144222SRS019787\_contig\_number\_contig-100\_2097.2097SRS020233\_contig\_number\_708SRS023971\_contig\_number\_contig-100\_6240.6240SRS024331\_contig\_number\_12793SRS024435\_contig\_number\_14449SRS048164\_contig\_number\_24018SRS049402\_contig\_number\_contig-100\_4598.4598SRS049712\_contig\_number\_contig-100\_3060.132834SRS049896\_contig\_number\_6145SRS050941\_contig\_number\_636SRS052027\_contig\_number\_14520SRS053214\_contig\_number\_20680SRS056519\_contig\_number\_contig-100\_923.100786SRS058770\_contig\_number\_19811SRS063040\_contig\_number\_38005SRS064557\_contig\_number\_contig-100\_15594.15594SRS064645\_contig\_number\_contig-100\_10457.10457SRS065504\_contig\_number\_contig-100\_11239.91463SRS077127\_contig\_number\_7767SRS077730\_contig\_number\_17263SRS098073\_contig\_number\_12390SRS1041112\_contig\_number\_contig-100\_4087.4088SRS1055056\_contig\_number\_contig-100\_4763.43736SRS142503\_contig\_number\_contig-100\_788.305560SRS143780\_contig\_number\_contig-100\_3105.197329SRS144183\_contig\_number\_contig-100\_16210.67711SRS147346\_contig\_number\_68074SRS148159\_contig\_number\_51656SRS893383\_contig\_number\_737
39
59620
species

2
1263021
species

SRS043667\_contig\_number\_contig-100\_15536.15536SRS104036\_contig\_number\_1278


SRS016335\_contig\_number\_contig-100\_935.183449
species
2292889
1
